# Quinine as a Prophylactic in Puerperal Fever

**Published:** 1849-04

**Authors:** 


*Quinine as a Prophylactic in Puerperal Fever.*—M. Leudet cites several cases which occurred during the prevalence of three different epidemics at Rouen, in which quinine, given in doses of five grains three times a day, and commenced with a few hours after delivery, seemed to exert a protective agency. On the third day, this quantity was diminished, and the drug left off about the sixth day. In those epidemics in which the disease commences very speedily after delivery, the quinine should be administered as soon as labor begins.—*L'Union Médicale.*—*Ibid.*

